# Post-pipeline headache after flow-diverting stenting for unruptured intracranial aneurysms: clinical, radiological findings, and proposed scoring system

**DOI:** 10.1007/s00415-026-13863-5

**Published:** 2026-05-27

**Authors:** Marina Romozzi, Matteo Palermo, Monica Ferrante, Federico Tosto, Antonio Funcis, Raffaele Turano, Luigi Francesco Iannone, Catello Vollono, Giovanni Frisullo, Paolo Calabresi, Iacopo Valente, Francesco D’Argento, Andrea Alexandre, Giuseppe Garignano, Alessandro Pedicelli

**Affiliations:** 1 Department of Neuroscience, Università Cattolica del Sacro Cuore, Rome, Italy; 2 Neurology Unit, Dipartimento di Neuroscienze, Organi di Senso e Torace, Fondazione Policlinico Universitario Agostino Gemelli IRCCS, Rome, Italy; 3 Radiology Unit, Ospedale S’Anna, Como, Italy; 4 Department of Biomedical, Metabolic and Neural Sciences, University of Modena and Reggio Emilia, Modena, Italy; 5 Radiology and Neuroradiology Unit, Dipartimento di Diagnostica per Immagini, Radioterapia Oncologica ed Ematologia, Fondazione Policlinico Universitario Agostino Gemelli IRCCS, Rome, Italy; 6 Department of Neuroscience, Giovanni Paolo II Hospital, Lamezia Terme, Italy

**Keywords:** Flow diverter, Stent, Aneurysm

## Abstract

**Background and objectives:**

Headache after flow diverter (FD) treatment for unruptured intracranial aneurysms (UIAs), historically referred to as post-pipeline headache, is increasingly recognized but remains poorly characterized and lacks standardized diagnostic criteria. We aimed to investigate the prevalence, clinical features, and predictors of post-pipeline headache and to internally validate a structured scoring system for this entity.

**Methods:**

In this single-center cohort study, we included consecutive adult patients undergoing FD treatment for UIAs between 2021 and 2025. Headache characteristics before and after treatment were assessed using structured interviews. A composite post-procedural headache score was developed to identify post-pipeline headache (*post-pipe score*). Content validity index (CVI) was calculated. Diagnostic performance was assessed with receiver operating characteristic (ROC) curve analysis against independent neurologist classifications. Multivariable logistic regression was performed to identify predictors of post-procedural headache.

**Results:**

Among 137 patients (mean age 58.3 ± 12.7 years; 19% male), post-pipeline headache occurred in 29 (21.2%), including 12 patients with new-onset headache and 17 with worsening of pre-existing headache. The scoring system demonstrated excellent content validity (CVI = 0.96) and good diagnostic discrimination across raters (AUC range 0.84–0.91), supporting a threshold ≥ 9 points to identify post-pipeline headache. Larger aneurysm neck size was independently associated with post-procedural headache (OR 1.47, 95% CI 1.04–2.07, *p* = 0.025), while dome size was also larger in patients with post-pipeline headache (*p* = 0.042).

**Discussion:**

Post-pipeline headache is a common clinical phenomenon affecting approximately one-fifth of patients following FD treatment for UIAs. Larger aneurysm neck size appears to be an independent predictor of post-pipeline headache, supporting a potential role for local vascular remodeling and inflammatory mechanisms.

**Supplementary Information:**

The online version contains supplementary material available at 10.1007/s00415-026-13863-5.

## Introduction

An unruptured intracranial aneurysm (UIA) is a relatively common condition, occurring in about 2–3% of the population [1]. Over the years, treatment options have progressed significantly, from early techniques, such as craniotomy and aneurysm wrapping to clipping, endovascular coiling, and, more recently, the use of flow diverters (FDs). Flow diverters are endovascular, low-porosity, stent-like devices deployed within the parent artery across the aneurysm neck, designed to modify local hemodynamics by reducing blood flow into the aneurysm sac, thereby promoting progressive intra-aneurysmal thrombosis and subsequent endothelialization across the neck, ultimately leading to parent vessel reconstruction and aneurysm exclusion from the circulation [2]. The introduction of FDs has greatly widened the scope of aneurysms suitable for endovascular therapy, making cases once thought difficult far more manageable [2].

The International Classification of Headache Disorders, 3rd edition (ICHD-3) classifies headache attributed to an unruptured saccular aneurysm among secondary headache disorders [3]. Diagnosis requires the presence of a newly developed headache in an individual with a documented unruptured aneurysm and clinical evidence suggesting a causal relationship. However, although headache is reported in about one-fifth of patients with unruptured cerebral aneurysms, whether this association is causal or incidental remains uncertain [3].

After treatment for a UIA, headache patterns can change: some patients experience improvement, others worsening symptoms, and some develop an entirely new type of headache [4, 5].

However, most available studies include heterogeneous treatment strategies, combining different flow diverters, stent-assisted coiling, and coiling alone, often in relatively small cohorts; consequently, headache outcomes are inconsistent across studies and remain difficult to interpret [4, 5].

Regarding FD specifically, the few available studies describe a condition named “post-pipeline headache,” referring to a new-onset or qualitatively different headache occurring after FD implantation for UIA. This pain may cause significant anxiety and discomfort and often leads to additional neuroimaging to exclude hemorrhagic complications or other causes [6, 7].

A deeper understanding of post-pipeline headache with its risk factors, natural history, and potential treatment responses could improve pre-procedural counseling and may guide future clinical trials aimed at reducing periprocedural complications [6, 7].

In the present study, we aimed to investigate headache status after pipeline stenting for intracranial aneurysms, detailing pain characteristics and prior headache history in a large cohort of individuals.

## Methods

### Study design and population

This ambispective study included patients who underwent endovascular treatment with FD between December 2021 and December 2025 at *IRCCS Policlinico Gemelli* (Rome, Italy). It combined retrospective extraction of procedural and imaging variables with prospective structured headache assessment during follow-up interviews.

Patients older than 18 years who provided written informed consent for the use of their clinical and radiological data for research purposes were included. Patients had at least 3 months of follow-up after the procedure. The 3-month cut-off was selected to identify headache persisting beyond the immediate post-procedural period and to reduce the risk of misclassifying transient procedure-related symptoms as persistent headache. Exclusion criteria comprised incomplete clinical or radiological data and potential confounding factors for headache, such as other neurological disorders or systemic diseases that could influence headache. A history of ruptured aneurysm or intracranial bleeding was also considered an exclusion criterion.

Clinical data were collected through telephone interviews or during in-person outpatient visits. For each patient, demographic information (age and sex), comorbidities, and treatments were recorded.

Considering T0 as the time of FD placement, the previous headache history was collected to identify primary headache disorders or secondary headache attributed to UIA according to the ICHD-3. These data were collected during phone interviews or in-person visits (M.R., A.F., R.T., and F.T.).

Headache characteristics were evaluated before and after T0, with follow-up assessments performed at 3 months and 12 months.

### Study variables

For patients with a history of primary headache disorders, the following characteristics were recorded: age at onset, headache side (unilateral, bilateral), accompanying symptoms (i.e., nausea, vomiting, photophobia, phonophobia), mean monthly headache frequency (MHDs), mean pain intensity assessed using the numeric rating scale (NRS, 0–10 scale), and the mean total number of analgesics (AMNs) and type of analgesics used during the 6 months preceding T0. Based on the features above, and according to ICHD-3, primary headaches were classified as migraine or tension-type headache. Finally, all available data on acute and preventive migraine treatments were collected. The same characteristics were collected at 3 and 12 months (post-T0 period).

For headache occurring after T0, patients were also asked to rate the similarity (in overall features) between their current headache and their usual primary headache using a subjective scale from 0 (very different) to 10 (identical).

All post-T0 headache characteristics were combined into a newly developed score designed to capture changes in headache features before and after FD placement, as described in Table 1.

**Table 1:** Scoring system for post-pipeline headache (post-pipe score)

Criterion	Domain	Operational definition	Score
A	Temporal relationship	Development of a new headache persistent for at least 3 months after flow diverter implantation	Required
B	Headache-naïve patients	In patients with no prior history of headache	Diagnostic (+ 9)
C			
1	Intensity change	Difference of 2–4 points on 0–10 NRS between pre- and post-procedural headache intensity	+ 2
		Difference of ≥ 4 points on 0–10 NRS between pre- and post-procedural headache intensity	+ 4
2	Lateralization changes with respect to aneurysm side	Contralateral → ipsilateral	+ 2
		Contralateral → contralateral	+ 0
		Ipsilateral → contralateral	+ 0
		Ipsilateral → ipsilateral	+ 0
3	Attack duration category change	Categories: < 4 h 4–24 h 24–72 h > 72 h	
		1 point assigned per category transition	+ 1
		5 points assigned per ≥ 2 category transition	+ 5
4	New-onset associated symptoms	Variation between pre- and post-pipeline in Vomiting Photophobia Phonophobia Visual disturbances	+ 1
		Nausea	+ 2
5	Headache frequency	A ≥ 50% increase in headache frequency and a minimum difference of 5 days between the pre- and post-procedural phases	+ 4
6	Subjective dissimilarity	Patient-reported comparison on 0–10 scale 8–10 = completely different 6–8 = markedly different	+ 4 (score ≥8) + 2 (score 6–8) + 0 (score <6)
D	Exclusion of alternative diagnoses	Headache not better explained by another primary headache disorder or secondary cause (ICHD-3)	Required
	Diagnostic threshold	A + D + B/C	≥ 9

Stenting-related variables were also recorded, including the type of endovascular treatment, duration of dual antiplatelet therapy (DAPT), and whether patients continued with mono antiplatelet therapy (MAPT) after DAPT and for how long. Additional variables included the administration of corticosteroids in the post-procedural period and the occurrence of aneurysm recanalization requiring re-intervention.

Two experienced neuroradiologists (A.P. and A.A.) (> 10 years of experience each) independently reviewed digital subtraction angiography (DSA) images and measured aneurysm dome and neck dimensions. In cases of measurement discordance, the mean value was used for the final analysis. Topographical information regarding aneurysm location was also collected.

The study conforms to the ethical guidelines of the 1975 Declaration of Helsinki, as reflected in a priori approval by the institution’s human research committee at. The study was reported according to the Strengthening the Reporting of Observational Studies in Epidemiology (STROBE) guidelines.

The research was approved by the Ethical Committee of Università Cattolica del Sacro Cuore (DEUTEROS study—ID 269599).

### Scoring system, internal validation, and content validity index

To identify patients who developed post-pipeline headache, two groups were considered, focusing on the timeframe 3 months after FD placement: those with new-onset headache and those with worsening of pre-existing headache.

The classification of new-onset headache was straightforward: patients who developed a headache that persisted for at least three months after the procedure, and that was not better explained by another primary or secondary headache disorder according to the ICHD-3, were classified as having post-pipeline headache.

For patients with a pre-existing history of migraine or other headache disorders, we investigated potential changes in headache characteristics after the procedure. To systematically evaluate these changes, a structured scoring system was used. Variations in intensity, frequency, and duration of headache attacks were considered the primary factors for detecting change. As individual episodes may vary in severity and length, these parameters were assessed according to their average pattern over time. In addition, patient-reported perception of worsening was taken into account; however, subjective perception alone was not considered sufficient to define worsening. Changes in associated symptoms were also evaluated as part of the assessment. The diagnostic threshold was established using specific changes in headache phenotype pre- vs post- intervention. For patients with new-onset headache, fulfillment of criteria A, B, and D was considered diagnostic. For patients with a pre-existing headache history, fulfillment of criteria A, C, D, and a score of ≥ 9 points was required. The total score was obtained by adding points assigned to relevant changes in headache characteristics after treatment, including intensity increase (+ 2 for 2–4 NRS points; + 4 for ≥ 4 points), lateralization change toward the aneurysm side (+ 2), attack-duration category transition (+ 1 for one category; + 4 for ≥ 2 categories), new or worsening of associated symptoms (+ 1; nausea + 2), ≥ 50% increase in headache frequency with at least 5 additional headache days (+ 4), and subjective dissimilarity from the baseline headache pattern (+ 2 for score 6–8; + 4 for score 8–10).

The detailed criteria used for classification are reported in Table 1*(post-pipe score).* In cases where a patient’s pre-existing primary headache showed only minimal worsening, insufficient to meet criteria for post-pipeline headache (a secondary headache disorder), this was considered within the range of normal fluctuations typical of primary headache disorders, particularly following an intervention. Therefore, such cases were classified as “no change”. Content validity was assessed by an expert panel of neurologists (headache specialists) and neuroradiologists (*n* = 11) (mean experience 7.7 years). Reviewers evaluated the relevance and clarity of sections A, B, C1–C6, and D using a 4-point Likert scale (1 = not relevant to 4 = highly relevant). The Item-level Content Validity Index (I-CVI) and Scale-level Content Validity Index (S-CVI/Average) were calculated to quantify the degree of expert consensus and instrument validity*.*

**Fig. 1:**
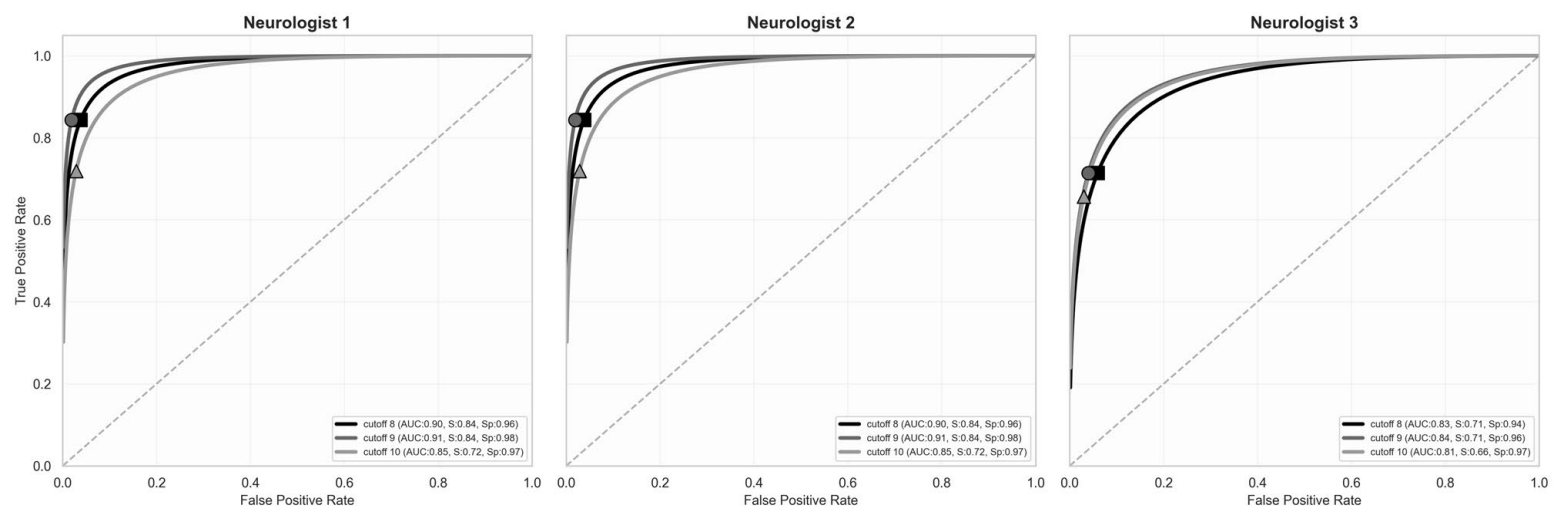
Receiver operating characteristic (ROC) curves showing the diagnostic performance of the scoring system (*post-pipe score*) as assessed independently by three neurologists. For each evaluator, three candidate cut-off values (8, 9, and 10) were tested. Cut-off = 9 showed the best overall balance between sensitivity and specificity across raters (Neurologist 1: AUC = 0.91, sensitivity = 0.84, specificity = 0.98; Neurologist 2: AUC = 0.91, sensitivity = 0.84, specificity = 0.98; Neurologist 3: AUC = 0.84, sensitivity = 0.71, specificity = 0.96), supporting its selection as the optimal threshold. The dashed diagonal line represents chance-level discrimination

To evaluate the diagnostic accuracy and performance of the scoring system, a comparative Receiver Operating Characteristic (ROC) curve analysis was performed. The performance of each cut-off was validated against three independent clinical classifications provided by three senior neurologists with expertise in headache disorders based on the clinical data (M.R., F.T., and C.V.). The authors classified patients as having post-pipeline headache or not. We exclusively plotted cutoffs 8, 9, and 10 as those were the best performing (Fig. 1).

For each validation, sensitivity, specificity, and area under the curve (AUC) were reported. To enhance visualization, ROC curves were generated using a binormal model fitting, and the threshold of ≥ 9 points was selected for the score to define post-pipeline headache. All analyses were performed using R (v4.6.0).

### Statistical analysis

Continuous variables were summarized as mean ± standard deviation or median (interquartile range) according to data distribution, while categorical variables were reported as counts and percentages. Normality of data distribution was assessed using the Shapiro–Wilk test. Since most variables showed non-normal distributions, non-parametric analyses were preferred. Comparisons between groups were performed using Fisher’s exact test for categorical variables. For continuous or ordinal variables, the Mann–Whitney *U* test was used as appropriate.

Group comparisons were performed between patients with and without post-pipeline headache, as well as between patients with new-onset headache and those with worsening pre-existing headache.

In a second step, a multivariate logistic regression analysis was conducted to identify predictors of post-pipeline headache, which was treated as a dichotomous outcome variable according to the cut-off score of 9 (yes/no). All relevant variables were initially assessed as potential predictors.

To reduce the risk of model overfitting, the baseline model (Model 0) was restricted to age and sex as covariates.

Statistical analyses were performed using JASP software (v.0.96.0; March 5th, 2026) and R (v4.6.0). A two-tailed *p* value < 0.05 was considered statistically significant.

## Results

### Overall cohort findings

A total of 137 patients underwent endovascular treatment for intracranial aneurysms between December 2021 and December 2025. The mean age of the cohort was 58.29 ± 12.70 years, 19.0% males.

The majority were located in the anterior circulation (86.1%). Mean aneurysm dome size was 10.90 ± 6.62 mm, with a mean neck size of 5.09 ± 2.53 mm and an average aspect ratio of 2.26 ± 1.08. Regarding treatment, coils (spirals) were used in 29 (21.2%) cases. Postoperative steroid therapy was administered in 88 (66.2%) patients during the postoperative course. These patients received dexamethasone 4–8 mg/day once daily for 3–7 days. All patients received DAPT initially, with a mean duration of 3.789 ± 1.99 months, followed by maintenance with MAPT in 107 (81.1%) patients for an average of 5.35 ± 3.61 months.

Overall, 88/133 patients (64.2%) had a history of a headache disorder prior to FD placement. Among these, 25 (28.4%) had a prior diagnosis of tension-type headache and 51 (57.9%) had migraine. Eighteen patients (18/88, 20.5%) were classified as having suspected secondary headache related to the aneurysm, defined according to ICHD-3 criteria. Among these patients, six had a pre-existing history of headache before the onset of the secondary form, including five with migraine and one with tension-type headache. Overall, 6 patients were on prophylactic treatment before intervention.

### Content validity index

The analysis demonstrated excellent content validity for the proposed scoring (*post-pipe score*), with an overall S-CVI/Ave of 0.96. Relevance scores averaged 3.49 (I-CVI range 0.82–1.0), while clarity scores averaged 3.55 (I-CVI range 0.91–1.0). Experts unanimously supported the proposed diagnostic threshold of ≥ 9 points (mean 3.64, 100% agreement) (Supplementary Figure). See also Fig. 1 for ROC curves.

### Headache outcome after FD

**Fig. 2:**
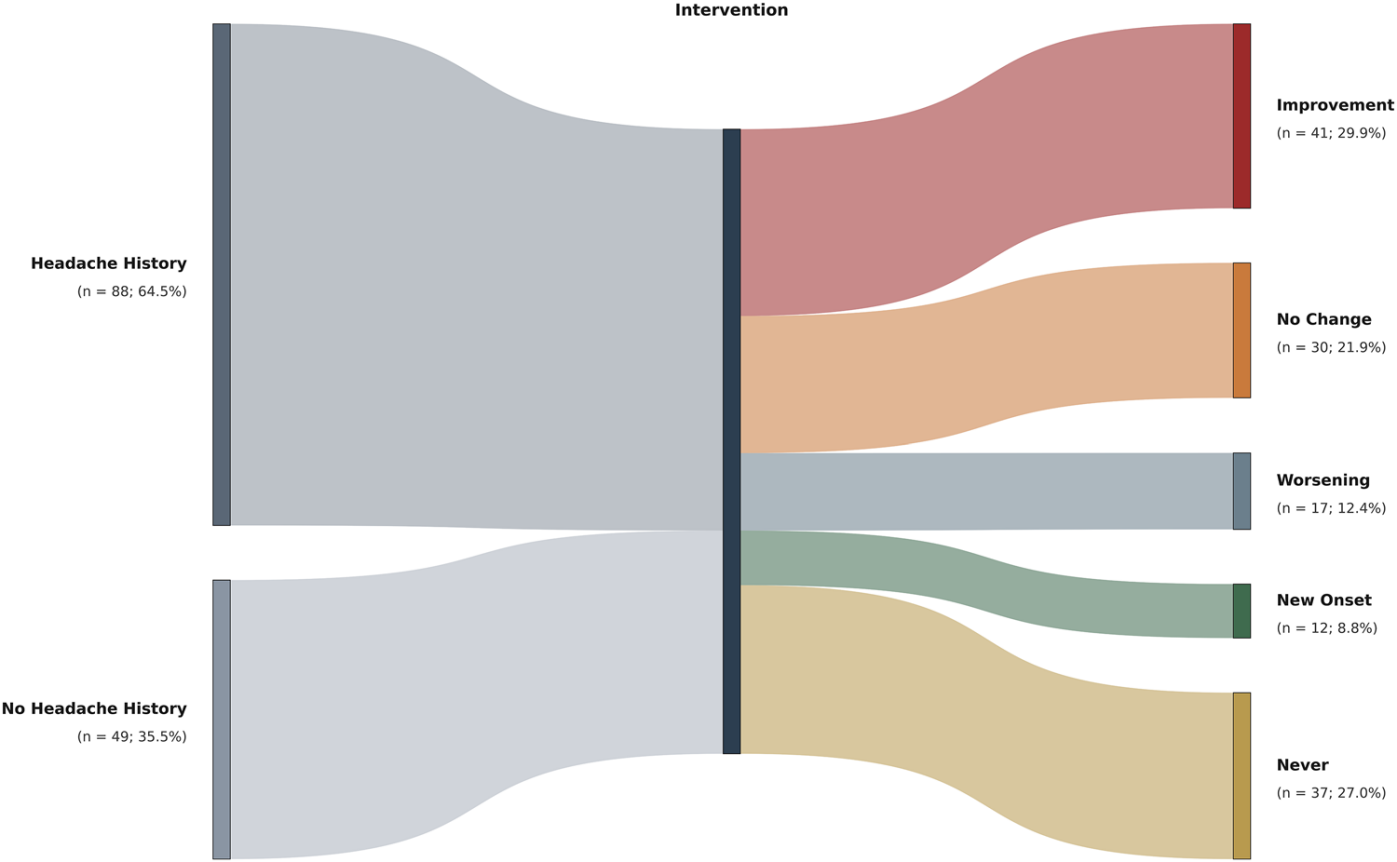
Sankey diagram illustrating changes in headache status after flow diverter (FD) placement. Patients who developed a headache after the procedure without a prior history were classified as having a new-onset headache. Patients with pre-existing headache who reported an improvement, regardless of changes in headache characteristics, were put in the “improvement” group. Patients reporting no differences compared with the pre-procedural period were labeled as “no change.” Patients without a prior history of headache who remained headache-free after the procedure were classified as “never”. Patients reporting worsening symptoms compared with the pre-procedural phase, including changes in headache features, were classified as “worsening”

Following FD placement, 41/137 patients (29.9%) reported improvement in their headache pattern, whereas 30/137 (21.9%) experienced no change from baseline. Overall, headache worsening was observed in 17/137 patients (12.4%), while 12/137 patients (8.8%) developed new-onset headache. Notably, 37/137 patients (27.0%) had never had a relevant headache before and after the procedure (Fig. 2). Among patients with a suspected secondary headache disorder related to the aneurysm, 3/18 (16.7%) worsened and developed a post-pipeline headache, while the others improved.

### Post-pipeline headache

After applying the inclusion and exclusion criteria, 108 patients were classified as non-post-pipeline headache and 29 as post-pipeline headache.

Among patients with no prior headache history, 24.4% (12/49) developed new-onset headache after treatment. Differently, among patients with a pre-existing headache history, 46.5% (41/88) reported an improvement, while 19.3% (17/88) experienced worsening. Mean *post-pipe score* of those with worse headache was 10.82 ± 1.944. Additionally, pre-operative pain distribution showed that the most common location was the frontal region (7/17, 41.2%), followed by diffuse pain (6/17, 35.3%) and orbicular/periorbital pain (2/17, 11.7%). Occipital pain and pain located on the same side as the aneurysm were each reported in 1 patient (5.8%). No patients reported pain on the side opposite to the aneurysm. Data on location were available for only 12 patients. The most frequent pain distribution was unilateral pain as the aneurysm in 4 patients (33.3%), followed by frontal pain in 4 patients (33.3%). Diffuse pain was reported in 3 patients (25.0%), while posterior pain occurred in 1 patient (8.3%). No patients reported orbicular/periorbital pain or pain on the side opposite to the aneurysm.

No significant differences were observed between the two groups (namely, post-pipeline headache and not) in terms of age, sex, aneurysm morphology, or aneurysm location. Similarly, when aneurysms were stratified according to anterior versus posterior circulation, no significant differences were found between groups.

Conversely, the aneurysm neck and dome size were statistically significant different between the two groups. The mean neck size was 4.83 ± 2.20 mm in the non-post-pipeline group and 6.03 ± 3.39 mm in the post-pipeline group (*p* = 0.043). Likewise, dome size was significantly larger in the post-pipeline group (13.68 ± 8.47 mm) compared with the non-post-pipeline group (10.14 ± 5.85 mm; *p* = 0.042). The type of endovascular treatment did not differ significantly between groups. Specifically, the use of adjunctive coils did not reach statistical significance. Similarly, no differences were observed between groups regarding duration of antiplatelet therapy or post-procedural steroid administration (Table 2).

**Table 2:** Comparison in demographic, clinical, and radiological characteristics of patients with and without post-pipeline headache

	Overall cohort (*n* = 137)	Non-post-pipeline headache (*n* = 108)	Post-pipeline headache (*n* = 29)	*p* value
Age, mean ± SD	58.29 ± 12.70	58.60 ± 12.38	57.11 ± 14.07	0.573
Sex (M), *n* (%)	26 (19.0)	23 (21.3)	3 (10.3)	0.185
Topography, *n* (%)				0.893
Vertebral-Basilar	14 (10.2)	13 (12.0)	1 (3.4)	
Vertebral-PICA	2 (1.5)	2 (1.9)	0 (0.0)	
Basilar-P1	2 (1.5)	1 (0.9)	1 (3.4)	
ACA				
A1–A2	1 (0.7)	1 (0.9)	0 (0.0)	
Acom	4 (2.9)	4 (3.7)	0 (0.0)	
A2–A3	5 (3.7)	3 (2.8)	2 (6.9)	
ICA				
Cervical	1 (0.7)	1 (0.9)	0 (0.0)	
Petrous	1 (0.7)	1 (0.9)	0 (0.0)	
Cavernous	13 (9.5)	10 (9.3)	3 (10.3)	
Ophthalmic	68 (49.6)	52 (48.1)	16 (55.2)	
Choroidal	1 (0.7)	1 (0.9)	0 (0.0)	
Pcom	18 (13.1)	14 (13.0)	4 (13.8)	
A1	2 (1.5)	1 (0.9)	1 (3.4)	
M1	1 (0.7)	1 (0.9)	0 (0.0)	
MCA				
M1-M2	2 (1.5)	1 (0.9)	1 (3.4)	
PCA				
P1–Pcom	1 (0.7)	1 (0.9)	0 (0.0)	
P2–P3	1 (0.7)	1 (0.9)	0 (0.0)	
Anterior (vs posterior circulation), *n* (%)	118 (86.1)	91 (84.3)	27 (93.1)	0.364
Aneurysm dimension (mm), mean ± SD				
Neck size	5.08 ± 2.53	4.83 ± 2.20	6.03 ± 3.39	**0.043**
Dome size	10.90 ± 6.62	10.14 ± 5.85	13.68 ± 8.47	**0.042**
Aspect ratio	2.25 ± 1.07	2.16 ± 1.15	2.33 ± 1.08	0.370
Endovascular details, *n* (%)				
Use of spirals	29/132 (22.0)	20/105 (19.0)	9/27 (33.3)	0.123
Post-implantation treatment details,				
Dual antiplatelet therapy duration (months), mean ± SD	3.77 ± 1.99	3.59 ± 1.34	4.48 ± 3.50	0.192
Mono antiplatelet therapy duration (months), mean ± SD	5.35 ± 3.61	5.57 ± 3.90	4.50 ± 2.01	0.405
Mono antiplatelet therapy (y/n), *n* (%)	107/132 (81.1)	84/106 (79.2)	23/26 (88.5)	0.230
Steroid (vs no), *n* (%)	88/133 (64.2)	66/106 (62.3)	22/27 (81.5)	0.070
Need for re-intervention (vs no), *n* (%)	4/96 (4.2)	2/75 (2.7)	2/21 (9.5)	0.207

P-value refers to non-post-pipeline headache vs post-pipeline headache. Data are presented as mean ± SD or n (%). When data were available only for a subset of patients, the number of available cases is reported.

*ACA* Anterior cerebral artery, *Acom* Anterior communicating artery, *Aspect ratio* Ratio between aneurysm height and neck width, *Basilar-P1* Basilar artery–P1 segment junction, *ICA* Internal carotid artery, *M1* First segment of the middle cerebral artery, *M1–M2* Junction between M1 and M2 segments of the middle cerebral artery, *MCA* Middle cerebral artery, *P1–Pcom* Junction between P1 segment and posterior communicating artery, *P2–P3* Junction between P2 and P3 segments of the posterior cerebral artery, *PCA* Posterior cerebral artery, *Pcom* Posterior communicating artery, *PICA* Posterior inferior cerebellar artery.

When the cohort of 29 patients with post-pipeline headache was further analyzed according to new-onset versus worsening headache, no significant differences were observed in dome or neck size between the two subgroups. Likewise, no other clinical or treatment-related variables differed significantly between these groups. Detailed results are reported in Supplementary Table.

Finally, after adjusting for age and sex, multivariate logistic regression analysis demonstrated that aneurysm neck size was independently associated with the development of post-pipeline headache (OR 1.47, 95% CI 1.04–2.07, *p* = 0.025). None of the other variables analyzed reached statistical significance (Table 3).

**Table 3:** Multivariate logistic regression analysis of post-pipeline headache, adjusted for age and sex

	*β* value	SE	Odds ratio (CI)	*p* value
Age	− 0.016	0.023	0.984 (0.9414–1.028)	0.475
Sex (male vs female)	− 1.152	0.916	0.316 (0.052–1.904)	0.209
Dome size	0.008	0.055	1.008 (0.904–1.123)	0.888
Anterior circulation (vs posterior)	1.725	1.182	5.611 (0.553–56.957)	0.145
Neck size	0.388	0.173	1.474 (1.049–2.070)	**0.025**
Dual antiplatelet therapy duration (months)	0.333	0.196	1.395 (0.951–2.047)	0.088
Mono antiplatelet therapy duration (months)	− 0.192	0.130	0.825 (0.639–1.064)	0.139
Partial thrombosis	− 3.736	2.233	0.024 (0.001–1.862)	0.093

CI Confidence interval, SE Standard error

At 12-month follow-up, data were available for 16 out of 29 patients (55.2%) who were classified as part of the post-pipeline group at 3 months, as the remaining patients either had not yet reached the required time frame or were lost to follow-up. Among the 8 patients in the new-onset group with available follow-up data, 5 (62.5%) reported persistent post-pipeline headache. In the worsening group, 7 patients (87.5%) continued to experience post-pipeline headache. For those in the worsening group with persistent headache at 12-month follow-up, the acute medications prescribed were acetaminophen/paracetamol (*n* = 3), ketoprofen (*n* = 2), ibuprofen (*n* = 1), and naproxen (*n* = 1). Among patients with new-onset headache after treatment and available follow-up data, management-reported therapies included: ketoprofen (*n* = 2), paracetamol/acetaminophen (*n* = 2), ketorolac (*n* = 1), and short-term corticosteroid use (*n* = 1). Two patients reported no need for medication during follow-up. No patient was given any vasoactive medications during this period.

## Discussion

In this large cohort of patients undergoing FD treatment for unruptured intracranial aneurysms, post-pipeline headache occurred in nearly one-fifth of patients. The main finding of our study is that aneurysm neck size was independently associated with the development of post-procedural headache, with dome size also larger in patients with post-pipeline headache.

Previous studies investigating headache after endovascular treatment of intracranial aneurysms have reported heterogeneous results, largely reflecting mixed procedural cohorts, small sample sizes, and limited characterization of pre-existing primary headache disorders [4, 8]. In a cohort of 44 patients treated with coiling, clipping, or liquid embolic agents, 37 had a prior history of headache, and most patients (68%) experienced improvement after treatment, whereas new or worsened headache was observed in approximately 9% of cases, a proportion comparable with that observed in the present study [4]. There are no comparative studies between headache outcomes comparing FD and conventional stents.

Regarding FD implantation, the prevalence of what has been termed post-pipeline embolization headache varies widely across studies, ranging from approximately 5% to 30%, depending on study design and headache assessment methods, with most reports lacking detailed clinical characterization [6, 9–11].

In a telephone survey of 88 patients treated with FD stents for UIA, 55% reported a new or different headache after the procedure, with truly de novo headaches in 19 patients. Pain was typically described as dull, sharp, or throbbing and ipsilateral to the treated aneurysm. Headache onset occurred on average 20 days after treatment, and 69% of patients were still symptomatic at a mean follow-up of 21.6 months. Younger age and a prior history of headache were identified as predictors of post-procedural headache [6].

In another cohort of 60 patients, 53.3% had a history of headache before treatment. After the procedure, 25% experienced complete headache resolution, whereas among those with persistent headache, frequency and severity decreased or remained stable in most cases. New-onset headache was reported in 18.3% of patients. Interestingly, this study suggested an association between larger aneurysm size and headache improvement [10]. While another study reported an association between larger aneurysm size and the development of post-procedural headache, our findings were consistent with these observations [12]. In particular, the association between aneurysm neck size and dome size with post-pipeline headache in a larger and more systematically characterized cohort provides further insight into the potential underlying pathophysiological mechanisms. One plausible explanation is a local inflammatory response triggered by device implantation and subsequent endothelial remodeling of the parent vessel. Experimental and clinical evidence support the role of vascular wall inflammation and endothelial activation in stimulating trigeminovascular afferents involved in intracranial pain transmission [13]. In addition, progressive thrombosis within the aneurysm sac after FD placement may contribute to local inflammatory processes that can trigger headache [14]. Larger necks are typically associated with wider surface of interaction between the device and the vessel wall, potentially enhancing local hemodynamic changes and endothelial responses and increasing the likelihood of trigeminovascular activation [15]. Although aneurysm morphology has been extensively investigated in relation to procedural outcomes, its role in post-procedural headache has been only marginally explored [16].

Another relevant issue is the relationship between pre-existing primary headache disorders and post-procedural headache. Migraine and tension-type headache are common in the general population and frequently reported in patients with intracranial aneurysms, making it challenging to distinguish worsening of a primary disorder from the emergence of a secondary headache [6, 17, 18]. To address this limitation, we adopted a structured scoring system to systematically evaluate changes in headache characteristics after treatment. This system demonstrated optimal content validity and was internally validated through independent scoring by neurologists.

From a clinical perspective, recognition of post-pipeline headache is important because these symptoms frequently lead to patient concern and additional diagnostic investigations [19]. Patients presenting with new headache after FD or stent placement often undergo urgent neuroimaging to exclude hemorrhagic complications; improved characterization of this condition may therefore help clinicians provide reassurance and reduce unnecessary imaging in the absence of warning signs [6, 7].

Future revisions of the ICHD should consider a more precise definition of this entity. Although the term post-pipeline headache is widely used and easily recallable, it refers to a specific device brand and therefore may not represent the most appropriate nomenclature for this condition [20].

This study has several strengths, including the inclusion of a homogeneous cohort treated exclusively with flow diverter devices, the longitudinal design, and the detailed characterization of headache features before treatment, with specific consideration to distinguishing primary from secondary headache disorders. In addition, the multiparametric scoring system used to define headache worsening allowed a more objective assessment of post-procedural changes. To our knowledge, this is the first study providing structured diagnostic criteria supported by content validity and ROC-based threshold validation for post-flow-diverter headache.

However, some limitations should be acknowledged. Although this was pre-planned, only patients treated with flow diverter devices were assessed, and therefore, we cannot determine whether similar findings would be observed with other types of stents. Changes in pain character were not recorded due to the lack of standardized documentation and the unreliability of retrospective patient recall. Additionally, although 81% of the cohort was female, the study included all consecutive patients treated during the study period, without any selection bias. This female predominance may be partly explained by the higher reported risk of aneurysm rupture in women, potentially leading neuroradiologists to recommend earlier intervention [21]. Finally, the retrospective assessment of pre-procedural headache characteristics may have been affected by recall bias, and not all patients completed the 12-month follow-up, limiting evaluation of long-term outcomes.

## Conclusion

Our findings support the existence of a clinically relevant secondary headache disorder following flow diversion treatment and identify aneurysm neck size as a potential predictor. Prospective studies with standardized headache assessment and larger cohorts are needed to clarify the underlying mechanisms and to identify patients at increased risk.

## Supplementary Information

Below is the link to the electronic supplementary material.

Supplementary file1 (DOCX 20 KB)

Supplementary file2 (PNG 74 KB)

## Data Availability

All data supporting the results of this study are included within the article and its supplementary material. Additional datasets generated and analyzed during the study are available from the corresponding author upon reasonable request.
