# Supplementary material for: Post-pipeline headache after flow-diverting stenting for unruptured intracranial aneurysms: clinical, radiological findings, and proposed scoring system
Source: J Neurol. 2026 May 27;273(6):345. doi: 10.1007/s00415-026-13863-5 (PMC13216145; doi:10.1007/s00415-026-13863-5)
Supplement: Supplementary file 1 — Supplementary file1 (DOCX 20 KB) [file 415_2026_13863_MOESM1_ESM.docx]

**Supplementary Table 1.** Univariate analysis comparing new-onset vs worsening types of post-pipeline headaches.

|  | | | **New onset (n=12)** | **Worsening (n=17)** | **p-value** |
| --- | --- | --- | --- | --- | --- |
| **Age** | | | 59.417±13.970 | 55.267±14.355 | 0.541 |
| **Sex (M), n (%)** | | | 0 (0.0) | 3 (17.6) | 0.246 |
| **Topography, n (%)** | | |  |  | 0.336 |
|  | Vertebral-Bailar | | 0 (0.0) | 1 (5.9) |  |
|  | Vertebral-PICA | | - | - |  |
|  | Basilar-P1 | | 0 (0.0) | 1 (5.9) |  |
|  | ACA | |  |  |  |
|  | | A1-A2 | - | - |  |
|  | | Acom | - | - |  |
|  | | A2-A3 | 1 (8.3) | 1 (5.9) |  |
|  | ICA | |  |  |  |
|  | | Cervical | - | - |  |
|  | | Petrous | - | - |  |
|  | | Cavernous | 1 (8.3) | 2 (11.8) |  |
|  | | Ophthalmic | 9 (75.0) | 7 (41.2) |  |
|  | | Choroidal | - | - |  |
|  | | Pcom | - | - |  |
|  | | A1 | 1 (8.3) | 0 (0.0) |  |
|  | | M1 | - | - |  |
|  | MCA | |  |  |  |
|  | | M1-M2 | 0 (0.0) | 1 (5.9) |  |
|  | PCA | |  |  |  |
|  | | P1-Pcom | 0 (0.0) | 4 (23.5) |  |
|  | | P2-P3 | - | - |  |
| **Anterior (vs Posterior Circulation), n (%)** | | | 12 (100.0) | 15 (88.2) | 0.498 |
| **Aneurysm dimension** | | |  |  |  |
|  | Neck | | 5.292±2.098 | 6.533±4.055 | 0.462 |
|  | Dome | | 11.233±5.752 | 15.412±9.765 | 0.478 |
|  | Aspect Ratio | | 2.129±0.713 | 2.481±1.285 | 0.451 |
| **Endovascular details, n (%)** | | |  |  |  |
|  | Use of Spirals | | 4/12 (33.3) | 5/15 (33.3) | 1.00 |
| **Post-Implantation Treatment details** | | |  |  |  |
|  | Dual antiplatelet therapy duration (months ± SD) | | 3.750±1.865 | 5.067±4.383 | 0.250 |
|  | Mono antiplatelet therapy duration (months ± SD) | | 4.364±2.461 | 4.636±1.567 | 0.452 |
|  | Mono antiplatelet therapy (y/n), n (%) | | 11/12 (91.7) | 12/14 (85.7) | 1.00 |
|  | Steroid (vs none), n (%) | | 9/12 (75.0) | 13/15 (86.7) | 0.628 |
| **Need for reintervention (vs none), n (%)** | | | 1/10 (10.0) | 1/11 (9.1) | 1.00 |
