# Supplementary figures and images for: Post-pipeline headache after flow-diverting stenting for unruptured intracranial aneurysms: clinical, radiological findings, and proposed scoring system
Source: J Neurol. 2026 May 27;273(6):345. doi: 10.1007/s00415-026-13863-5 (PMC13216145; doi:10.1007/s00415-026-13863-5)

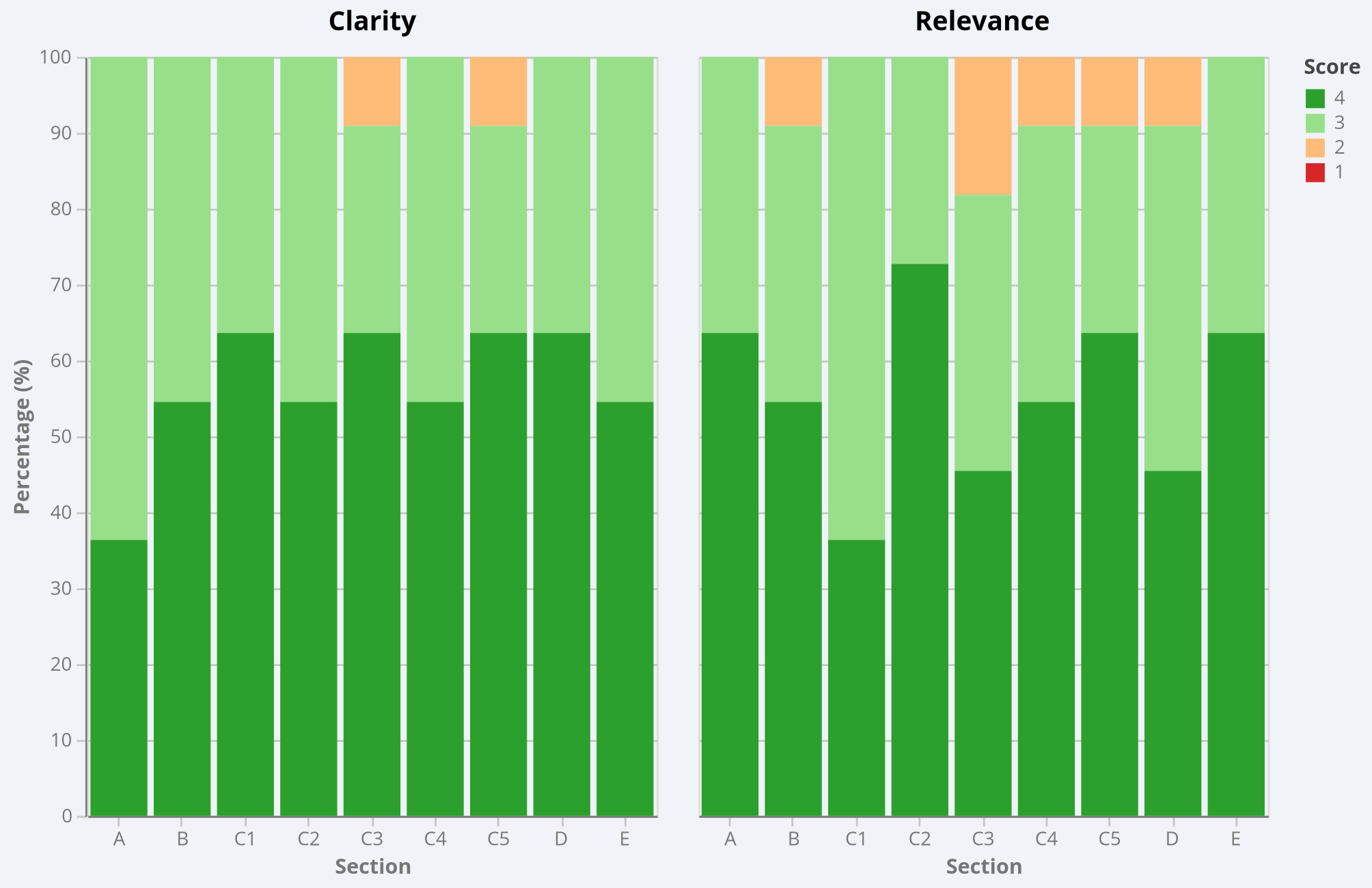

Supplement: Supplementary file 2 — Supplementary file2 (PNG 74 KB) [file 415_2026_13863_MOESM2_ESM.png]
